# Visual prognosis of submacular hemorrhage secondary to age-related macular degeneration: A retrospective multicenter survey

**DOI:** 10.1371/journal.pone.0271447

**Published:** 2022-07-21

**Authors:** Naomi Inoue, Aki Kato, Takashi Araki, Takeshi Kimura, Takamasa Kinoshita, Fumiki Okamoto, Tomoya Murakami, Yoshinori Mitamura, Taiji Sakamoto, Akiko Miki, Yoshihiro Takamura, Hisashi Matsubara, Hiroki Tsujinaka, Fumi Gomi, Tsutomu Yasukawa

**Affiliations:** 1 Department of Ophthalmology and Visual Science, Nagoya City University Graduate School of Medical Sciences, Nagoya, Japan; 2 J-CREST (Japanese Clinical Retina Study) Group, Kagoshima, Japan; 3 Department of Ophthalmology, Hyogo College of Medicine, Nishinomiya, Japan; 4 Department of Ophthalmology, Sapporo City General Hospital, Sapporo, Japan; 5 Department of Ophthalmology, Faculty of Medicine, University of Tsukuba, Tsukuba, Japan; 6 Department of Ophthalmology, Institute of Biomedical Sciences, Tokushima University Graduate School, Tokushima, Japan; 7 Department of Ophthalmology, Kagoshima University Graduate School of Medical and Dental Sciences, Kagoshima, Japan; 8 Department of Surgery, Division of Ophthalmology, Kobe University Graduate School of Medicine, Kobe, Japan; 9 Department of Ophthalmology, Faculty of Medical Sciences, University of Fukui, Yoshida, Japan; 10 Department of Ophthalmology, Mie University Graduate School of Medicine, Tsu, Japan; 11 Department of Ophthalmology, Nara Medical University School of Medicine, Kashihara, Japan; National Yang-Ming University Hospital, TAIWAN

## Abstract

**Purpose:**

To investigate the clinical features, treatment options, and visual outcomes of submacular hemorrhage (SMH) secondary to neovascular age-related macular degeneration (nAMD).

**Design:**

A retrospective, observational case series.

**Methods:**

Setting: Multicenter institutional setting. Patient Population: A total of 127 patients (127 eyes; 88 men, 39 women; (mean age, 74.2 years)) diagnosed with AMD-associated SMHs exceeding 2 disc diameters involving the fovea. Observation: The AMD types, previous treatments, treatment options, anatomic findings, and best-corrected visual acuity (BCVA) were assessed. Main Outcome Measures: Clinical features, treatment options, and visual outcomes of SMHs secondary to nAMD.

**Results:**

Thirty-two eyes had typical AMD, 94 eyes polypoidal choroidal vasculopathy (PCV), and one eye retinal angiomatous proliferation. Eighty-five eyes were treatment-naïve; 42 eyes were treated previously: anti-vascular endothelial growth factor (VEGF) therapy (n = 26), photodynamic therapy (n = 3), and combined therapy (n = 13). Treatment of SMHs included vitrectomy (36 eyes), pneumatic displacement (49 eyes), and anti-VEGF monotherapy (42 eyes). The final BCVA improved significantly in treatment-naïve cases from 0.86 to 0.62 logarithm of the minimal angle of resolution (logMAR) unit (Snellen equivalent from 20/145 to 20/83) and from 0.80 to 0.56 (Snellen equivalent from 20/126 to 20/73) in PCV cases. Meanwhile, the BCVA logMAR values improved from 1.15 to 0.75 (Snellen equivalent from 20/283 to 20/112) and from 0.87 to 0.63 (Snellen equivalent from 20/148 to 20/85) in eyes that underwent vitrectomy or pneumatic displacement, respectively. In eyes with BCVAs between 20/133 to 20/40 at SMH onset, the final VA in the pneumatic displacement group was better than in the anti-VEGF monotherapy group. One eye had a retinal detachment and 1 eye had a macular hole in the vitrectomy group, and 5 eyes had a vitreous hemorrhage in the pneumatic displacement group.

**Conclusions:**

The recommended treatment for SMHs secondary to nAMD exceeding 2 disc area and with BCVA below 20/40 is vitrectomy or pneumatic displacement for visual improvement.

## Introduction

Neovascular age-related macular degeneration (nAMD) is a major cause of visual loss in elderly individuals in developed countries, and the incidence in Japan has been increasing in recent years [1]. Anti-vascular endothelial growth factor (VEGF) therapies such as intravitreal ranibizumab (IVR) (Lucentis^®^, Genentech Inc., South San Francisco, CA) and aflibercept (Eylea^®^, Regeneron Pharmaceuticals Inc., Tarrytown, NY) and photodynamic therapy (PDT) are effective [2–6]. However, during the disease course, extensive submacular hemorrhages (SMHs) sometimes occur even during treatment. In particular, polypoidal choroidal vasculopathy (PCV), the most common form of the disease in the Japanese population [7], can cause severe SMHs more frequently than other types of nAMD [8, 9]. Since persistent SMHs cause irreversible visual impairment, various interventions have been performed to shift the hemorrhages away from the submacular space. Typical treatments include intravitreal injection of expansile gas with or without tissue plasminogen activator (tPA) [10–13], vitrectomy [14–18], and anti-VEGF therapy [19]. Nevertheless, while there are several treatment strategies for SMHs, the optimal strategy remains undetermined. The objective of this multicenter study was to investigate the clinical features, treatment options, and visual outcomes of SMHs secondary to nAMD.

## Methods

### Study design and ethics

The current study was a retrospective, multicenter, observational case series of patients who were examined at 10 institutions participating in the Japanese Clinical Retina Study (J-CREST) Group. This study was stated after approved from the institutional review boards of the following study sites: Nagoya City University, Hyogo College of Medicine, Sapporo City General Hospital, University of Tsukuba, Tokushima University, Kagoshima University, Kobe University, University of Fukui, Mie University, and Nara Medical University School of Medicine. This study was conducted in compliance with the ethical guidelines of the Declaration of Helsinki. All patients provided written informed consent and permission to use the clinical data in this study. This study was registered with UMIN (UMIN000036698) retrospectively.

### Patients

One hundred twenty-seven eyes of 127 consecutive patients (88 men, 39 women; mean age, 74.2 ± 9.2 years) diagnosed with a SMH associated with nAMD between April 2015 and September 2018 were included. The inclusion criteria were eyes with a greatest linear dimension of SMHs exceeding 2 disc diameters and subfoveal involvement that had undergone any kind of intervention, had been followed for at least 1 month after the intervention, and had been followed at least 2 months after SMH onset. The exclusion criteria were eyes with a recurrent SMH or eyes with history of other vitreoretinal diseases such as retinal detachment, diabetic retinopathy, retinal vein occlusion, and uveitis.

The medical data collected included the follow-up periods, lens status, spherical equivalent, axial lengths, AMD subtypes, treatment history, disease duration, complications, and sizes of and treatments for the SMH.

**Fig 1:**
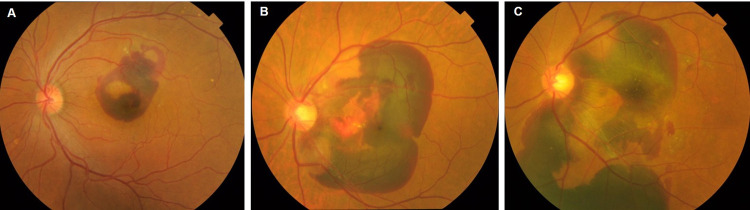
Categorization of SMH size. The image shows the sizes of the SMHs. A: within 5 DA; B: within the arcade vessel, and C: beyond the arcade vessel.

The ophthalmologic examination including measurement of the best-corrected visual acuity (BCVA), fundus imaging by color fundus photography or ultra-widefield fundus imaging, and optical coherence tomography (OCT) performed at least at baseline, 1 month after intervention, and at the final visit. The SMH size was assessed on the color fundus photographs or ultra-widefield fundus images. A massive SMH that was difficult to measure was considered to be 50 disc areas (DA). We also classified the SMHs roughly into within 5 DA, within the arcade vessel, and beyond the arcade vessel (Fig 1). The central retinal thickness (CRT), defined as the distance between the inner retinal surface and the outer portion of the hyperreflective line corresponding to the retinal pigment epithelium (RPE) on B-scan OCT images, was measured.

### Statistical analysis

The BCVAs at baseline, month 1, and the final visit and the best BCVA after treatment of the SMH were compared with that at the onset of the SMHs using a paired t-test. The differences of the BCVAs between eyes with and without previous treatment and between eyes with PCV and typical AMD were compared using an unpaired t-test. The differences in the BCVAs among treatment types were compared using Bonferroni’s test. The impact of the time from the last intervention to the onset of SMH and the size of the SMH on the treatment options were evaluated using the Steel-Dwass test. Fisher’s exact test was used to analyze the SMH size. Pearson’s correlation coefficient was used to analyze the correlation between variable parameters. *P* < 0.05 was considered statistically significant. Microsoft Excel software (Microsoft Corporation, Redmond, WA) was used for the statistical analyses.

## Results

### Patient baseline characteristics

The clinical characteristics of the study patients are shown in Table 1. Eighty eight (69%) of 127 patients were men. Ninety-four (74%) eyes had PCV. Eighty-five eyes (67%) were treatment-naïve. Sixty-seven of the 85 eyes (79%) had not undergone an ophthalmologic examination before the onset of SMH. Sixty-five of 94 (69%) eyes with PCV were treatment-naïve as were 19 of 32 (59%) eyes with typical AMD.

**Table 1:** Baseline characteristics and initial treatment for SMH of 127 eyes of 127 patients.

Parameter		
Gender	Male/female	88/39
Age (years) (±SD)	Mean	74.2 ± 9.2
	Range	45–95
Follow-up period (months) (±SD)	Mean	18.1 ± 12.1
	Range	2–47
Lens status (%)	Phakia	87 (68)
	Pseudophakia	38 (30)
	Aphakia	2 (2)
Spherical equivalent (D) (±SD)	Mean	0.20 ± 2.59 (n = 124)
	Range	-9.0 - +9.0
Axial length (mm) (±SD)	Mean	23.7 ± 1.3 (n = 52)
	Range	20.6–27.3
BCVA (logMAR) (±SD)	Mean	0.86 ± 0.56
CRT (μm) (±SD)	Mean	583 ± 232
Subtype (%)	Typical AMD	32 (25)
	PCV	94 (74)
	RAP	1 (1)
Previous treatment history (%)	Treatment naïve	85 (67)
	Anti-VEGF monotherapy	26 (21)
	PDT monotherapy	3 (2)
	Anti-VEGF + PDT combined therapy	13 (10)
SMH size (%)	Within 5 disc area	36 (28)
	Within arcade vessel	52 (41)
	Beyond arcade vessel	38 (30)
	Unmeasurable	1 (1)
Initial treatment for SMH (%)	Anti-VEGF monotherapy	42 (33)
	Pneumatic displacement	49 (39)
	With/without tPA	35/14
	Displaced by SF_6_/C_3_F_8_	41/8
	Vitrectomy	36 (28)
	No tamponade/air/SF_6_/C_3_F_8_/SO	3/9/17/1/6
	Intravitreal/subretinal/without tPA	4/20/12
Time from initial treatment to SMH onset (%)	Within 1 week	55 (43)
	Within 2 weeks	32 (25)
	More than 2 weeks	39 (31)
	Unknown	1 (1)

AMD = age-related macular degeneration; PCV = polypoidal choroidal vasculopathy; RAP = retinal angiomatous proliferation; BCVA = best-corrected visual acuity; logMAR = logarithm of the minimum angle of resolution; CRT = central retinal thickness; VEGF = vascular endothelial growth factor; PDT = photodynamic therapy; SMH = submacular hemorrhage; D = diopters; tPA = tissue plasminogen activator; SF_6_ = sulfur hexafluoride; C_3_F_8_ = octafluoropropane; SO = silicone oil; SD = standard deviation.

**Fig 2:**
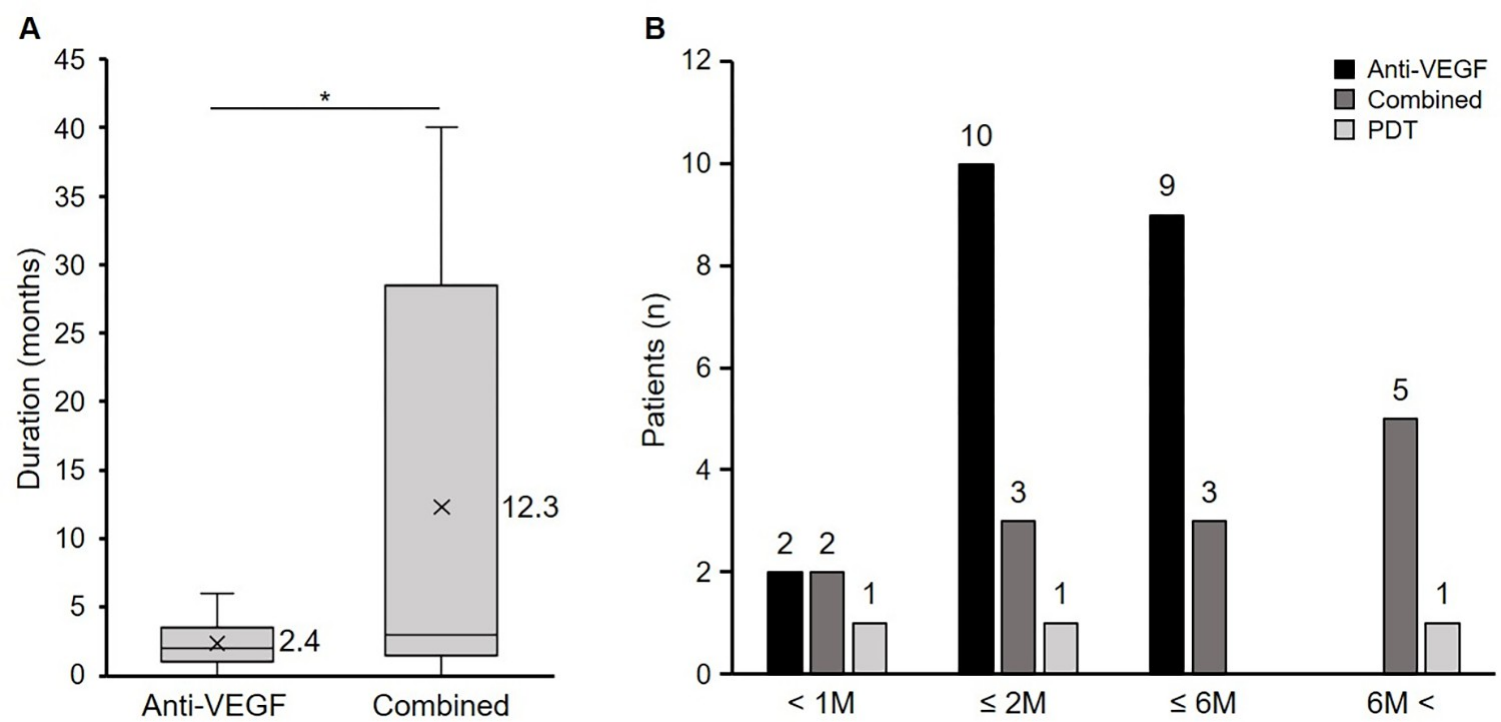
Time from the last intervention to the onset of the SMHs. A: The mean time from the last treatment to the onset of the SMHs was significantly shorter in the anti-vascular endothelial growth factor (VEGF) monotherapy group than in the combined group (*P* < 0.01 unpaired t-test). B: The anti-VEGF monotherapy group had more patients with a shorter duration to the onset of the SMHs. Combined = combined therapy with anti-VEGF and photodynamic therapy; PDT: photodynamic monotherapy. M = months.

Forty-two (33%) eyes had undergone previous treatments for nAMD. Among them, the mean time from the onset of nAMD to the onset of the SMHs was 4.3 years. Until the SMHs developed, 39 eyes received anti-VEGF therapy (20 eyes intravitreal aflibercept injection (IVA), 4 eyes IVR injection, 1 eye intravitreal bevacizumab (IVB) [Avastin^®^, F. Hoffmann-La Roche, Ltd., Basel, Switzerland] injection, 12 eyes IVA and IVR, 1 eye IVB and IVA, and in 1 eye the treatment was unknown. An average of 6.1 injections was administered. PDT was applied in 16 eyes, i.e., once in 13 eyes, and 4, 5, and 7 times in 1 eye each. The mean duration from the last treatment to the onset of SMH was significantly shorter in the anti-VEGF monotherapy group than in the combined therapy group (*P* < 0.01, unpaired t-test) (Fig 2A). SMHs developed within 2 months in 12 of 21 eyes treated with anti-VEGF monotherapy and in five of 13 eyes in the combined therapy group (Fig 2B).

The SMHs were 5 DA or smaller in 36 (28%) eyes, larger than 5 DA but within the arcade vessels in 52 (41%) eyes, and larger beyond the arcade vessels in 38 (30%) eyes (Table 1).

Three main types of initial treatments for the SMHs were administered: anti-VEGF monotherapy, pneumatic displacement, and vitrectomy (Table 1). Of 49 eyes that underwent pneumatic displacement, 35 (71%) eyes were treated with tPA, and of the 36 eyes with vitrectomy, 24 (67%) eyes were treated with tPA. No correlation was found between AMD subtype and treatment choice. Of the 36 eyes that underwent vitrectomy, 12 eyes were pseudophakia and 24 eyes were phakia. Twenty eyes underwent cataract surgery combined with vitrectomy. The duration from the onset of SMH to treatment was within 1 week in 55 (43%) eyes, within 2 weeks in 32 (25%) eyes, and longer than 2 weeks in 39 (31%) eyes; in one eye, the point at which the SMH developed was unknown (Table 1). The times to initiation of the initial treatment for SMH did not differ significantly among the treatment groups.

Regarding complications, in the vitrectomy group, 1 eye had a retinal detachment, 3 eyes had vitreous hemorrhages, and 1 eye had a macular hole that developed intraoperatively. On the other hand, in the pneumatic displacement group, 3 eyes with vitreous hemorrhages required vitrectomy and 2 eyes with vitreous hemorrhages did not require surgery. No serious complications were reported in the anti-VEGF monotherapy group.

**Fig 3:**
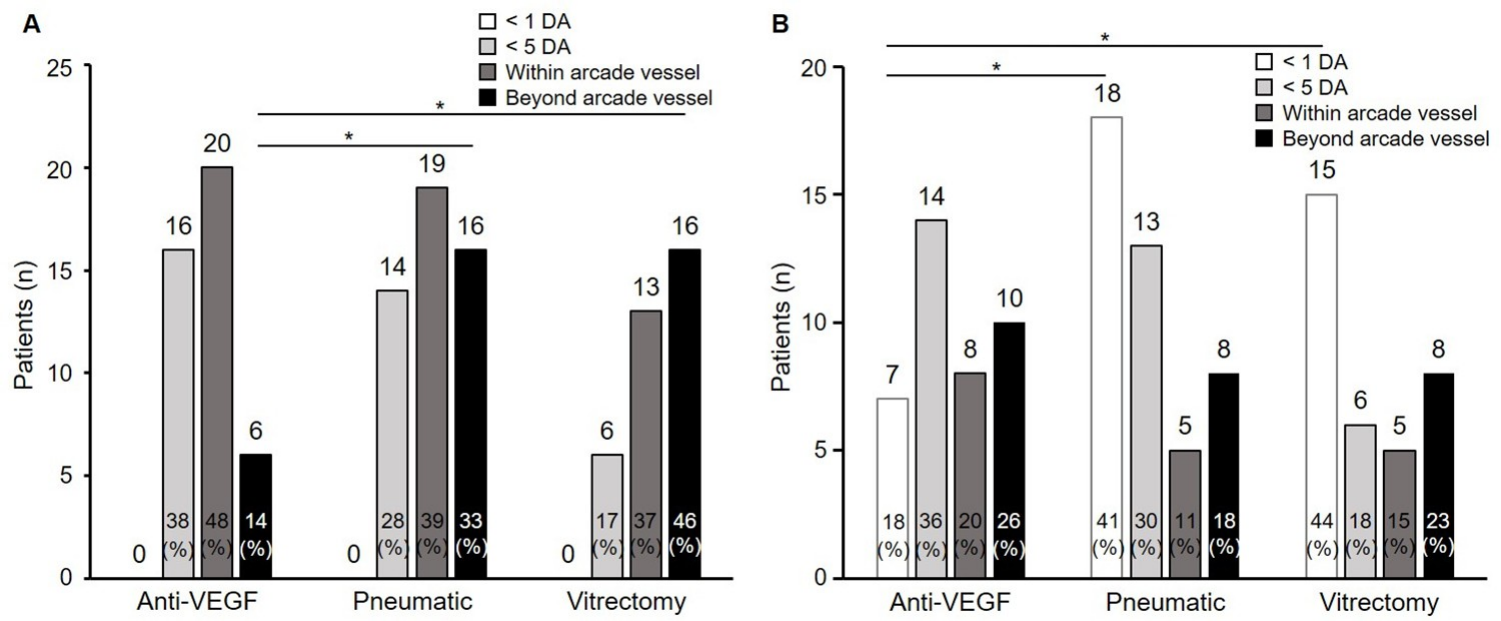
The sizes of the submacular hemorrhages (SMHs) based on the initial treatment before and 1 month after the initial treatment. A: The percentage of larger SMHs was higher in the vitrectomy group than in the anti-vascular endothelial growth factor (VEGF) monotherapy and pneumatic displacement groups (*P < 0.01 Fisher’s exact test). B: The percentages of patients with a SMH reduction within 1 disc area (DA) were significantly higher with pneumatic displacement and vitrectomy than with anti-VEGF monotherapy (*P* < 0.01, Fisher’s exact test). Pneumatic = pneumatic displacement.

The SMH sizes and the initial treatments are shown in Fig 3A. The mean size was 13.1 DA in the anti-VEGF monotherapy group, 16.8 DA in the pneumatic displacement group, and 23.5 DA in the vitrectomy group. The SMHs in the vitrectomy group were significantly larger than in the anti-VEGF monotherapy group (*P* < 0.05 Steel-Dwass test), and the ratio of the larger SMHs was higher in the vitrectomy group than in the anti-VEGF monotherapy and pneumatic displacement groups (*P* < 0.01 Fisher’s exact test).

The SMH sizes 1 month after the initial treatment are shown in Fig 3B. The ratio of eyes in which the SMH decreased below 1 DA was significantly higher in the pneumatic displacement (18 eyes, 41%) and vitrectomy (15 eyes, 44%) groups than in the anti-VEGF monotherapy group (7 eyes, 18%) (*P* < 0.01, Fisher’s exact test).

### Visual outcomes

**Fig 4:**
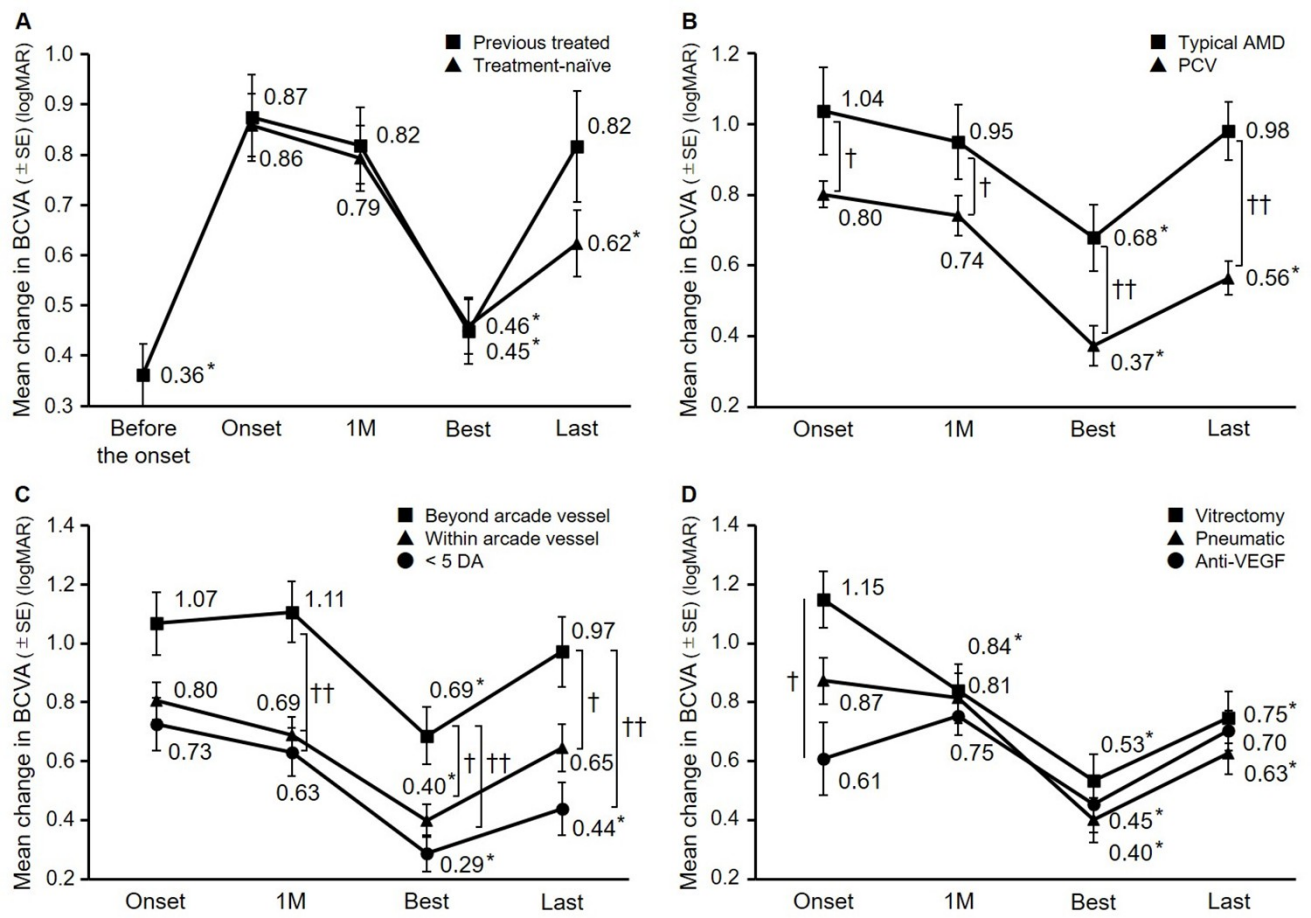
The changes in the mean Best-Corrected Visual Acuity (BCVA). A: In the treatment-naïve cases, the final VA improved significantly compared to the onset of the submacular hemorrhages (SMHs) (*P < 0.01, paired t-test), while in the previously treated cases, the VA deteriorated significantly as a result of the SMHs and did not improve after treatment. B: In eyes with polypoidal choroidal vasculopathy (PCV), the final VA improved significantly compared to that at the onset of the SMH (*P < 0.01, paired t-test), in eyes with typical age-related macular degeneration (AMD), the final VA was unchanged from that at the onset of the SMH. The mean BCVA in eyes with PCV was significantly better than in eyes with typical AMD at all time points (†*P* < 0.05, ††*P* < 0.01 unpaired t-test). C: Regarding all SMH sizes, the VA improved once, while only the SMHs smaller than 5 disc areas (DA) maintained the VA improvement (*P < 0.01, paired t-test), The BCVA in eyes with SMHs that extended beyond the arcade vessel was worse compared with other groups at all time points after intervention. (†*P* < 0.05, ††*P* < 0.01 Bonferroni test). D: In eyes treated with vitrectomy or pneumatic displacement, the BCVA improved at the final visit but remained unchanged in eyes treated with anti-VEGF monotherapy (*P* < 0.01, paired t-test). The VAs at the onset of SMHs were significantly better in eyes with treated with anti-VEGF monotherapy than vitrectomy. The final VAs did not differ significantly among the three groups. Best = at the visit with the highest BCVA recorded; Last = at the last visit; SE = standard error; M = month; Pneumatic = pneumatic displacement; logMAR = logarithm of the minimum angle of resolution.

Fig 4 shows the changes of the logarithm of the minimum angle of resolution (logMAR) BCVA. In the treatment-naïve cases, the best and last BCVAs improved significantly compared to that at the onset of the SMHs (*P* < 0.01, paired t-test), while in the previously treated cases, relatively good VA was maintained until just before the SMHs developed, at which time the VA deteriorated significantly and did not improve after treatment (Fig 4A).

In the eyes with PCV, the best and final BCVAs improved significantly compared to that at the onset of SMH (*P* < 0.01, paired t-test), while in eyes with typical AMD, the BCVA improved initially and then deteriorated and the significant improvement was lost with the onset of the SMHs. The mean BCVAs in eyes with PCV were significantly better at each time point than in eyes with typical AMD (*P* < 0.05 and *P* < 0.01, respectively, by the unpaired t-test) (Fig 4B).

Despite the SMH size, the BCVA improved initially (*P* < 0.01, paired t-test). However, in the group with SMHs larger than 5 DA, the significance of the BCVA improvement was lost, while the group with SMHs of 5 DA or smaller maintained the visual improvement (*P* < 0.01, paired t-test) (Fig 4C). In eyes with SMHs that extended beyond the arcade vessels, the BCVA at the onset of SMH was worse than in eyes with a SMH of 5 DA or smaller and eyes with SMHs within the arcade vessels at any time point after intervention (*P* < 0.05, Bonferroni’s test).

Vitrectomy or pneumatic displacement improved the mean BCVA at the last visit. However, anti-VEGF therapy did not improve the final BCVA (*P* < 0.01, paired t-test), because the BCVA at the onset of SMH in the anti-VEGF therapy group was the best among the groups (significantly better than in the vitrectomy group: *P* < 0.05, Bonferroni’s multiple comparison test). There were no significant differences in the final BCVAs among the groups (Fig 4D). There were also no significant differences in the final BCVAs between eyes with and without tPA in the vitrectomy group and the pneumatic displacement group.

**Fig 5:**
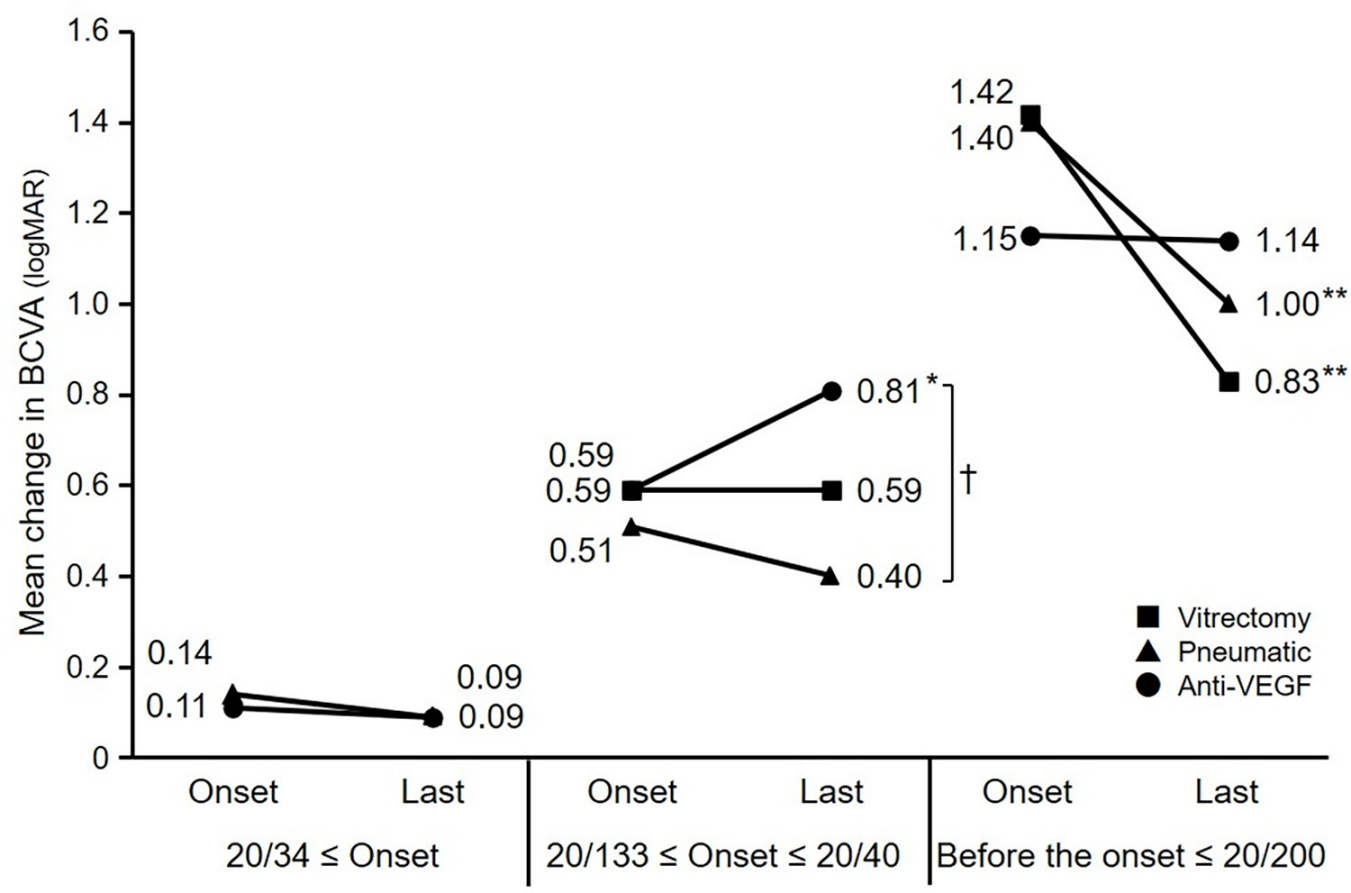
The changes in the mean best-corrected visual acuity (BCVA) based on the BCVA at the onset. In the group with a VA of 20/34 or more at the onset of the submacular hemorrhages (SMHs), there are no differences in the final VAs and between the anti-vascular endothelial growth factor (VEGF) monotherapy group and the pneumatic displacement group. In the group with VAs from 20/134 to 20/40 at the onset of the SMHs, the final VA in the pneumatic displacement group is 0.40, which is significantly better than in the anti-VEGF monotherapy group (*P* < 0.05, Bonferroni’s test), and in the anti-VEGF monotherapy group, the final VA decreased (*P* < 0.05, paired t-test). In the groups with VAs of 20/200 or less at the onset of the SMHs, there is no difference in the final VAs among the three groups. The final VAs improved in the vitrectomy group and the pneumatic displacement group (**P < 0.01 paired t-test). Pneumatic = pneumatic displacement; logMAR = logarithm of the minimum angle of resolution.

Fig 5 shows the BCVAs at the onset of the SMHs and the final visit. In the group with a BCVA better than 20/34 at the onset of the SMH, 10 eyes were treated with anti-VEGF monotherapy, six eyes with pneumatic displacement, and one eye with vitrectomy. There was no difference in the final BCVAs between the anti-VEGF monotherapy group and the pneumatic displacement group. The group with BCVAs ranging from 20/133 to 20/40 at the onset of the SMHs included 22 eyes treated with anti-VEGF monotherapy, 20 eyes with pneumatic displacement, and 10 eyes with vitrectomy. The final BCVA in the pneumatic displacement group was 0.40, which did not differ significantly from 0.59 in the vitrectomy group, but was significantly better than 0.81 in the anti-VEGF monotherapy group (*P* < 0.05, Bonferroni’s test). In the anti-VEGF monotherapy group, the final BCVA deteriorated (*P* < 0.05, paired t-test). The groups with BCVAs of 20/200 or worse at the onset of the SMHs included 10 eyes treated with anti-VEGF monotherapy, 22 eyes with pneumatic displacement, and 25 eyes with vitrectomy. While there were no differences in the final BCVA among the three groups, the final BCVAs improved significantly in the vitrectomy group and pneumatic displacement group compared with the BCVA at the onset of the SMHs (*P* < 0.01, paired t-test). There was no difference between vitrectomy and pneumatic displacement in the final BCVA.

### Correlations between the final VA and each parameter

Table 2 shows that the final logMAR BCVA was correlated positively with the SMH size, the BCVA at the onset of the SMHs, the CRT 1 month after the initial treatment, and AMD subtype, while there was no correlation between the CRT at the onset of the SMHs with the final BCVA (*P* < 0.05, Pearson’s rank correlation coefficient).

**Table 2:** Correlations between the final visual acuity and each parameter.

	BCVA at Last Visit (logMAR)	
	rs	*P*
SMH size at onset	0.264	0.006
BCVA at onset	0.361	0.0001
CRT at onset	0.096	0.325
CRT at 1 month	0.383	0.0001
AMD subtype	0.356	0.014

rs = Pearson’s rank correlation coefficient; BCVA = best-corrected visual acuity; logMAR = logarithm of the minimum angle of resolution; SMH = submacular hemorrhage; CRT = central retinal thickness; AMD = age-related macular degeneration.

## Discussion

SMH is a serious complication that causes an acute visual loss with the potential for permanent visual impairment in eyes with nAMD [20–22]. Subretinal blood causes early retinal photoreceptor disintegration and subsequent cell death [23]. The blood clot causes fibrin-mediated tractional damage within the retinal laminations [23, 24]. Therefore, to reduce damage to the photoreceptors and RPE, SMHs should be displaced from the subfoveal space as soon as possible, and various treatment approaches have been attempted. In the current study, the J-CREST Group collected data on SMHs associated with nAMD and retrospectively reviewed the findings, including the treatment history, before, at the onset of, and after intervention for the SMHs.

We focused initially on the treatment history. Twenty-one of the 26 eyes that had been treated with anti-VEGF monotherapy had a history of regular visits before the onset of SMH. Interestingly, 12 of 21 eyes treated with anti-VEGF monotherapy had a SMH within 2 months and 10 of 12 eyes had a SMH during the second month after the anti-VEGF injection. In contrast, only five of 13 eyes treated with combined therapy had a SMH within 2 months. These results suggested that regular, especially bimonthly anti-VEGF dosing does not necessarily suppress SMHs. In addition, in eyes with a history of treatment, the VA deteriorated significantly as a result of the SMHs and did not improve after treatment. On the other hand, 67% of eyes were treatment-naïve and their visual prognosis was better. Regarding disease type and SMH size, PCV had a better visual prognosis than typical AMD, and the smaller the SMHs were, the better the visual prognosis. Small SMHs associated with PCV in the treatment-naïve cases were more likely to be associated with improved VA regardless of the treatment.

Although there were no clear criteria for the treatment choice at any of the participating institutions, there was a tendency for clinicians to choose anti-VEGF monotherapy for small SRHs and pneumatic displacement or vitrectomy for large SRHs. Pneumatic displacement or vitrectomy successfully shifted the SMHs from the macula in more than 40% of cases and maintained the postoperative visual improvement, as needed, with additional treatments. This suggested that shifting the SMHs away from the macula at an early stage may prevent macular damage from the hemorrhage, as previously suggested [10–12, 15, 16].

In the current study, no differences were seen in the visual outcomes between eyes with or without adjunctive use of tPA in cases of pneumatic displacement and vitrectomy. However, because many institutions used tPA based on previous reports [10, 11, 15, 16], it is difficult to discuss the impact of tPA on visual outcomes based on the current results because the number of cases in which tPA was not used was smaller than those in which tPA was used.

The current study evaluated possible factors associated with the final VA. The worse the VA and the larger the SMH were at their onset, the worse the final VA was. In contrast, no relationship was seen between the CRT at the onset of the SMHs and the final VA. This may be because in eyes with larger SMHs, pneumatic displacement or vitrectomy tended to be selected and successfully shifting the SMHs away from the macula. In contrast, the thicker the CRT after 1 month of treatment, the worse the final vision was.

The current study also focused on the VA at the onset of the SMHs. In the eyes with relatively good VA, anti-VEGF monotherapy or pneumatic displacement was the initial treatment for SMHs, but there was no difference in the visual prognoses between the two groups, suggesting that the VA could be maintained even with anti-VEGF monotherapy in eyes with small SMHs and without visual deterioration. However, for patients with VAs between 20/133 and 20/40, various treatment options were chosen, among which pneumatic displacement had the best visual prognosis followed by vitrectomy and anti-VEGF monotherapy. The anti-VEGF monotherapy resulted in a decreased final BCVA. The results suggested that eyes with moderate visual disturbance should be treated with pneumatic displacement or vitrectomy to shift the SMHs away from the macula. However, there was no difference between vitrectomy and pneumatic displacement in the final BCVA. In the case of pneumatic displacement, no serious complications were observed. This suggested that pneumatic displacement may be a better choice for simpler procedures.

In contrast, in eyes with VAs of 20/200 or lower, vitrectomy or pneumatic displacement tended to be performed and achieved displacement of SMHs and somewhat improved BCVA. Nevertheless, the visual prognosis for eyes with VAs of 20/200 or less was not good with any treatment. Therefore, currently available treatment options should be chosen carefully with consideration of the patient age, daily activities, patient wishes, presence or absence of vitreous hemorrhage, and the status of the fellow eye. The surgical approaches to treat massive SMHs with severe visual loss may need further refinement.

The current study had some limitations. This study was retrospective and the treatment strategies regarding the initial treatment itself and additional treatments differed according based on the discretion of the collaborating institutions and attending physicians. A randomized, prospective study is needed. Nevertheless, this multicenter study collected cases of SMHs associated with nAMD, which are not experienced frequently in a single institution. The study provided significant information on the recent characteristics of SMHs and current treatment trends in Japan and warrants future studies.

In conclusion, anti-VEGF monotherapy effectively suppresses choroidal neovascularization; it has less of an effect on shifting SMHs. In eyes with good VA at the onset of SMHs and those with small SMHs, anti-VEGF monotherapy may maintain the VA. However, in eyes with large SMHs, pneumatic displacement or vitrectomy is recommended. Especially in eyes with BCVAs between 20/40 and 20/133 and moderate-sized SMHs, pneumatic displacement can achieve concomitant visual improvement.

## Supporting information

S1 Data (XLSX)
